# An End-to-End CRSwNP Prediction with Multichannel ResNet on Computed Tomography

**DOI:** 10.1155/2024/4960630

**Published:** 2024-06-06

**Authors:** Shixin Lai, Weipiao Kang, Yaowen Chen, Jisheng Zou, Siqi Wang, Xuan Zhang, Xiaolei Zhang, Yu Lin

**Affiliations:** ^1^ College of Engineering Shantou University, Shantou 515063, China; ^2^ Department of Otolaryngology Second Affiliated Hospital of Shantou University Medical College, Shantou 515041, China; ^3^ Department of Radiology Second Affiliated Hospital of Shantou University Medical College, Shantou 515041, China

## Abstract

Chronic rhinosinusitis (CRS) is a global disease characterized by poor treatment outcomes and high recurrence rates, significantly affecting patients' quality of life. Due to its complex pathophysiology and diverse clinical presentations, CRS is categorized into various subtypes to facilitate more precise diagnosis, treatment, and prognosis prediction. Among these, CRS with nasal polyps (CRSwNP) is further divided into eosinophilic CRSwNP (eCRSwNP) and noneosinophilic CRSwNP (non-eCRSwNP). However, there is a lack of precise predictive diagnostic and treatment methods, making research into accurate diagnostic techniques for CRSwNP endotypes crucial for achieving precision medicine in CRSwNP. This paper proposes a method using multiangle sinus computed tomography (CT) images combined with artificial intelligence (AI) to predict CRSwNP endotypes, distinguishing between patients with eCRSwNP and non-eCRSwNP. The considered dataset comprises 22,265 CT images from 192 CRSwNP patients, including 13,203 images from non-eCRSwNP patients and 9,062 images from eCRSwNP patients. Test results from the network model demonstrate that multiangle images provide more useful information for the network, achieving an accuracy of 98.43%, precision of 98.1%, recall of 98.1%, specificity of 98.7%, and an AUC value of 0.984. Compared to the limited learning capacity of single-channel neural networks, our proposed multichannel feature adaptive fusion model captures multiscale spatial features, enhancing the model's focus on crucial sinus information within the CT images to maximize detection accuracy. This deep learning-based diagnostic model for CRSwNP endotypes offers excellent classification performance, providing a noninvasive method for accurately predicting CRSwNP endotypes before treatment and paving the way for precision medicine in the new era of CRSwNP.

## 1. Introduction

Chronic rhinosinusitis (CRS) is a common ear, nose, and throat disease. It has attracted much attention due to its high prevalence (approximately 10%), high recurrence rate (approximately 50%), and high healthcare costs (approximately $33 billion/year) [1–4]. Currently, European and Chinese guidelines classify chronic rhinosinusitis (CRS) into chronic rhinosinusitis with nasal polyps (CRSwNP) and chronic rhinosinusitis without nasal polyps (CRSsNP) [5, 6]. It has been found that there are significant geographic and racial differences in the number of eosinophils within nasal polyps in patients with CRSwNP. For Caucasians in the West, eosinophilia predominates in the tissues of patients with CRSwNP, with a proportion of approximately 80% [5]. However, in East Asia, such as China, Japan, and South Korea, the eosinophilic endotype is less than 50% in cases of CRSwNP [7–10]. Therefore, a related study classified CRSwNP into two endotypes, eCRSwNP and non-eCRSwNP, and found that the two have different patterns of inflammation and tissue remodeling and have very different responses to corticosteroid therapy [9–14]. This suggests that different CRSwNP endotypes require different treatment strategies. Therefore, an in-depth understanding of CRSwNP endotypes is essential to achieve precise treatment.

Currently, biopsies, the “gold standard” for diagnosing CRS endophenotypes, are performed preoperatively but do not reflect the overall inflammation of the disease, which may lead to increased misdiagnosis. Sinus computed tomography (CT) is an indispensable test for assessing disease severity and evaluating outcomes and can also be used to predict CRS endotypes and to develop a personalized and precise surgical strategy for patients with CRS [15, 16]. Previous studies have often used the LM (Lund-Mackay) scoring system and the GOSS (global osteitis scoring scale) osteitis scoring system to differentiate CRS endotypes [17, 18]. However, such shallow prediction models are prone to introduce subjective interference and are limited by the difficulty of the human eye in capturing microscopic features [19–21].

With the continuous development of machine learning technology, the technical research on the combination of deep learning and CT images has achieved certain results in the medical field, which can be used to assist clinicians in diagnosing related diseases and reduce the burden of doctors [22, 23]. As there are differences in the phenotypes of sinus CT images of patients with different CRSwNP endotypes, and deep learning has a powerful image feature extraction capability, we constructed a network model for predicting CRSwNP endotypes based on sinus CT. We hope that this network architecture can efficiently distinguish eCRSwNP and non-eCRSwNP patients based on the sinus CT images of CRSwNP patients, thus reducing the misdiagnosis rate of clinicians and promoting the development of precision diagnosis and treatment of CRSwNP, and realizing the provision of individualized and precise treatment for patients with different subtypes of CRSwNP to provide some help. In this study, we collected multiview sinus CT images to construct a dataset and constructed a multichannel network based on the ResNet network. Distinguishing from the study that used single-section sinus CT images to input neural network to predict CRS endotypes, our model design used axial, coronal, and sagittal three sinus CT images as network inputs. It is able to obtain more effective information from sinus CT images, which results in a superior classification performance index in predicting CRSwNP endotypes and provides more favorable conditions for clinicians to develop precise treatment plans.

## 2. Dataset and Methods

### 2.1. Dataset

This study included 192 patients of CRSwNP, who were treated at the Second Affiliated Hospital of Shantou University Medical College between 2020 and 2022. The inclusion criteria were as follows: meeting the diagnostic criteria and treatment guidelines for international CRS [5], no history of nasal surgery, normal liver and kidney function, and no oral corticosteroids or macrolide antibiotics taken within one month prior to treatment. The exclusion criteria were as follows: age under 18, fungal rhinosinusitis, posterior nasal polyps, nasal and sinus tumors, primary ciliary dyskinesia, cystic fibrosis, no prior sinus CT scan, and no prior sinus surgery.

After performing a spiral CT scan of the venous sinuses, we used the PACS (picture archiving and communication systems) workbench to copy all the sinus CT image data for each patient and processed them using the DICOM (digital imaging and communications in medicine) image format. These data included axial, coronal, and sagittal planes. Subsequently, the raw data were converted to PNG format using MATLAB tools. In order to establish a multichannel feature fusion classification model, we classified 22,265 CT images collected from 192 patients with CRSwNP, of which 72 were eosinophilic eCRSwNP and 120 were non-eCRSwNP. In the modeling process, we packaged the CT images in a single axial, coronal, and sagittal CT image as a group. In total, 7,036 data sets were obtained. Finally, we divided the 7,036 sets of data into training set, validation set, and test set in the ratio of 3 : 1 : 1 for further data processing and model building.

### 2.2. ResNet18 Network Architecture

**Figure 1:**
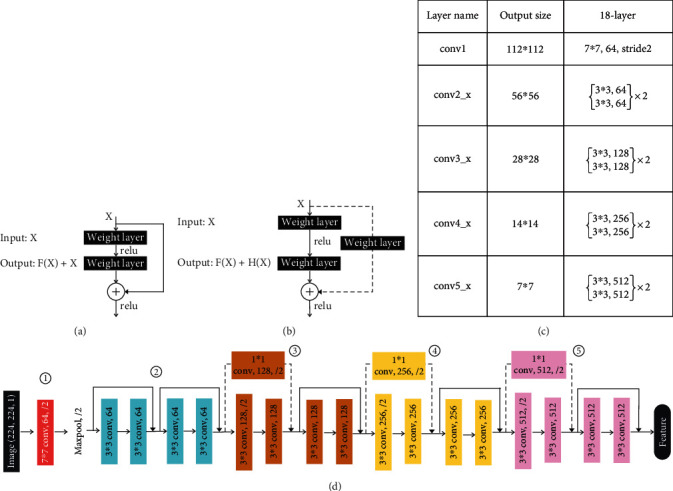
ResNet18 architecture. The dashed paragraphs (a) and (b) are the two connection structures of the residual block. (c) records the parameters of each convolutional block of the ResNet18 network, and (d) is a schematic of the ResNet18 architecture.

For classification tasks, the ResNet18 architecture is used to build a multichannel network for the feature extraction [25]. The ResNet18 architecture mainly consists of five convolutional modules, as shown in Figure 1. Convolutional module 1 consists of only one layer, while convolutional modules 2, 3, 4, and 5 all have two sets of convolutions. In modules 2, 3, 4, and 5, one set of convolutions contains two layers of convolutional layers and the convolutional groups are connected using residual connections, where module 2 is connected using only the architecture of residual module (a), while modules 3, 4, and 5 all use both (a) and (b) architectures. The residual module (b) is used to solve the inconsistency of the output size for different convolutional modules, so it is necessary to join 1 × 1 convolutional layers in the (b) branch connection to ensure that the feature module sizes match.

The residual module proposes two mapping connections; the first one is identity mapping, which is the branch line of the module (a) in Figure 1, *X* in the equation. The second one is residual mapping, which is the main straight line of the module (a) in Figure 1, and *F*(*X*) in the equation. This structure is proposed mainly because if there is only one channel for backpropagation, the successive products will make the gradient disappear as the number of network layers increases. If two channels exist, it can be turned into the form of summation, which avoids the gradient vanishing and enables researchers to build deeper network architectures to improve the model learning performance. And as images are a kind of data rich in learnable parameters, the ResNet architecture would be well suited for image classification tasks. Therefore, without image annotation, we utilized ResNet18 to predict endotype categories for CRSwNP patients with an accuracy of 95.9%.

### 2.3. Multichannel Fusion Deep Learning Network

**Figure 2:**
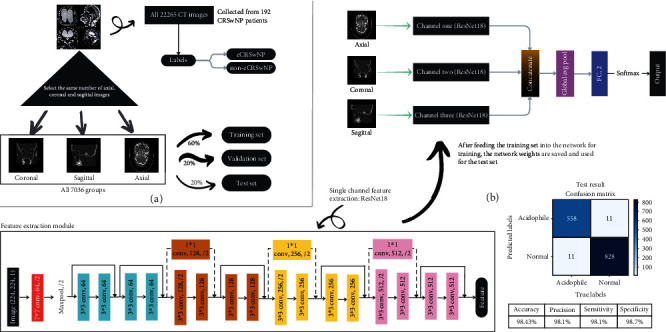
Experimental framework diagram. (a) The acquisition division of the dataset and (b) the architecture diagram of the multichannel feature fusion model with the test set experimental results.

Inspired by Siamese Network and Triplet Network [26, 27], we found that images from multiple layers and perspectives can provide more useful information to the network and thus improve the performance of the model. Therefore, we designed a multichannel feature adaptive fusion network architecture that combines information from multiple layers based on the ResNet18 architecture, as shown in Figure 2. We first grouped the established datasets for training, validation, and testing. Then, we train our model based on the ResNet18 architecture. The inputs to the model include the corresponding CT images in axial, coronal, and sagittal views. The three branch networks output feature maps which are aggregated into a fully connected dense layer. Finally, this layer outputs the endotype categories of CRSwNP patients and the performance of the deep learning model is evaluated using a confusion matrix.

### 2.4. Performance Evaluation

We evaluated the classification performance of the model using the confusion matrix and receiver operator characteristic (ROC) curve. With the confusion matrix, the predictive accuracy, sensitivity, and specificity of the model can be calculated, where accuracy is calculated as (true positive samples + true negative samples)/(all samples). Sensitivity is calculated as (true positive sample)/(true positive sample + false negative sample), while specificity is calculated as (true negative sample)/(false positive sample + true negative sample). In addition, the area under the ROC curve (AUC) can also be used to measure classifier performance. To evaluate the performance of the ResNet18 network and the multichannel fusion model, we also trained a single-channel model for comparison using axial view CT images as input.

## 3. Results

### 3.1. Comparison between Single-Channel and Multichannel Models

**Figure 3:**
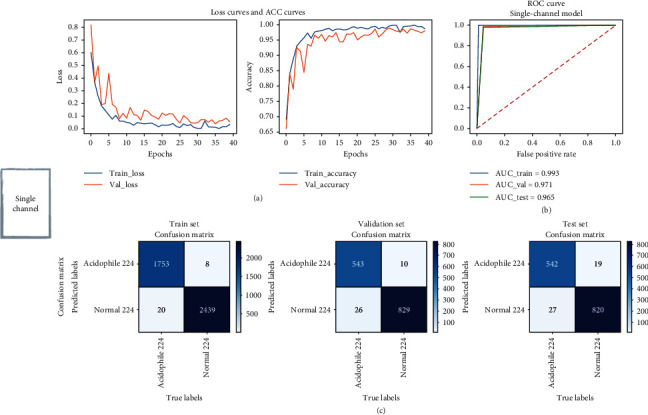
(a) Loss and accuracy curves, (b) ROC curves, and (c) confusion matrices for the single-channel CRSwNP endotype prediction models.

**Figure 4:**
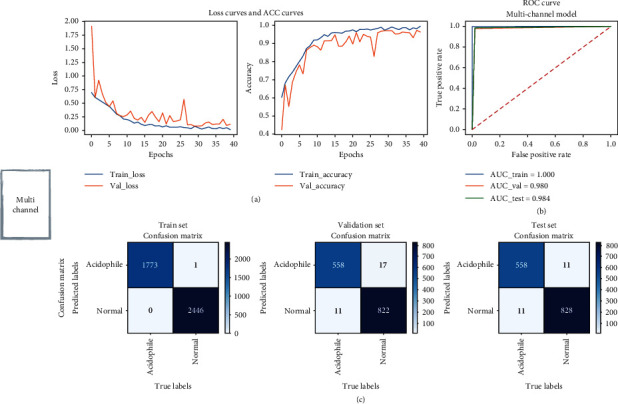
(a) Loss and accuracy curves, (b) ROC curves, and (c) confusion matrices for the multichannel CRSwNP endotype prediction models.

To illustrate the performance of the proposed model, we first evaluated a single-channel model trained using only axial view CT images. The model achieved AUCs of 0.993, 0.971, and 0.965 in the training, validation, and test sets, respectively. Its accuracy in the validation and test sets was 0.974 and 0.967, sensitivity was 0.954 and 0.953, and specificity was 0.988 and 0.977, respectively. For the multichannel model, the AUC, accuracy, and sensitivity in the training set reached 1. In the validation and test sets, the AUC was 0.980 and 0.984, respectively, while the accuracy was 0.980 and 0.984. In addition, the sensitivity was the same at 0.981, while the specificity was 0.980 and 0.987, respectively. The ROC curves and confusion matrices of the single-view and multiview models in the training, validation, and test sets are shown in Figures 3 and 4. The results show that using the multichannel model can achieve better performance in patient classification of CRSwNP.  Table 1 summarizes the comparison of the performance of single-channel and multichannel models.

### 3.2. Evaluate Model Performance on Two Levels

To further validate the performance of the model and make the results closer to the clinical diagnostic significance, we used two methods to obtain the results. The first method is based on the patient level. For each patient, it is assumed that Np is the number of all CT images of a patient, while Nrec is the image number of a patient that has been successfully classified by the model. The score for a patient can be calculated using the following equation:

(1) $$\begin{array}{c} \text{Patient score}=\frac{\text{Nrec}}{\text{Np}}. \end{array}$$

The recognition rate of all patients can be calculated using the following formula:

(2) $$\begin{array}{c} \text{Patient recognition rate}=\frac{\sum \text{P}\text{atient score}}{\text{Total number of patients}}. \end{array}$$

The second method is based on the level of images, without considering the patient's information. We calculate the number of all CT images in the test set—Nall, and the number of all correctly classified CT images—Nrec. The image recognition rate can be calculated using the following equation:

(3) $$\begin{array}{c} \text{Image recognition rate}=\frac{\text{Nrec}}{\text{Nall}}. \end{array}$$

According to the definition of Equation (2), CT images of individual patients were extracted from the original test set and predicted using the pretrained model weights. In the end, we obtained CT images of 172 patients with CRSwNP, including 66 cases of eosinophilic eCRSwNP and 106 cases of non-eCRSwNP. The prediction of these images yielded the results shown in Table 2, in which the patient identification rate was 0.976. Although it was a decrease from the result of 0.984 calculated by Equation (3), it was still efficient in screening the category of patients with CRSwNP.

## 4. Discussion

Chronic sinusitis with nasal polyps has a high recurrence rate and is expensive to treat, and there is a lack of precise treatment. The intrinsic type is a key factor in the accurate treatment of the disease. In terms of pharmacologic therapy, corticosteroids and doxycycline have been shown to be effective in eosinophilic CRS with nasal polyps (eCRSwNP), whereas macrolide antibiotics are effective in noneosinophilic CRSwNP (non-eCRSwNP). Surgical approaches aimed at preserving the sinus mucosa are more appropriate for non-eCRSwNP than for eCRSwNP [19, 24, 28, 29]. Therefore, different endotypes require different treatment regimens. Timely diagnosis of these endotypes is essential for the development of accurate treatment strategies.

Currently, there are relatively few studies on the diagnosis of CRSwNP endotypes based on machine learning combined with sinus CT, mostly based on the LM scoring system and the GOSS (global osteitis scoring scale) osteitis scoring system. Meng et al. used the LM scoring scale to predict CRSwNP endotypes and found that the CT score ratio (E/M ratio) of the sieve sinus and maxillary sinus was an effective predictor of eCRSwNP compared with other clinical data, with a predictive index ROC of 0.938, a sensitivity of 94.2%, and a specificity of 89.6% [20]. A GOSS-based CRS endotype prediction study found that the sieve osteitis index could be used for eCRS diagnosis, with a predictor ROC of 0.690, a sensitivity of 62%, and a specificity of 71% when its value was >4.5 [21, 30]. Although this means of prediction has some CRSwNP endotype prediction performance, it consumes a lot of time and energy to count the data because it uses semiqualitative and semiquantitative data, and its results are susceptible to subjective factors.

Combined with the arrival of the big data era, sinus CT data are large and easily accessible, providing a natural database for neural network training. Therefore, constructing a sinus CT database to train neural networks for automatic diagnosis of CRS endotype prediction has gradually become the mainstream of current research. Recently, Hua et al. applied U-net to construct a sinus CT-based CRS endotype prediction model and achieved 76.2% and 89.3% accuracy in image endotype labeling and patient endotype labeling prediction, respectively. Although this study coincided with the new period, it did not categorize the patients in detail but simply classified them into eCRS and non-eCRS and did not differentiate whether the patients were accompanied by nasal polyps or not, and its prediction accuracy still has room for improvement due to the limitations of U-net depth and single-channel data width.

Therefore, we further developed a deep learning model to achieve microscopic deep feature learning on sinus CT images to accurately predict CRSwNP endotypes and distinguish between eCRSwNP and non-eCRSwNP. Firstly, we constructed a high-precision database, which contained only multiview sinus CT images of patients with CRSwNP, from which we excluded images of patients with CRSsNP. Then, we input these data into a multichannel ResNet network for training, which was finally validated in a test set and found to achieve good prediction results. Compared with the single-channel network model, the multichannel model utilizes more CT image information, which makes it show better performance, while the single-channel model can only mine CT image information from a single viewpoint, and thus, the prediction effect will be reduced. These results indicate that our model has great potential for CRSwNP endotype classification diagnosis. It shows great potential in improving diagnostic efficiency and reducing radiologists' workload. It is conceivable that a tool based on multiview deep learning could be used to assist radiologists in quickly and accurately identifying endotypes in CRSwNP patients. In addition, it can effectively differentiate eCRSwNP patients from non-eCRSwNP patients based on preoperative sinus CT images, which may help in preoperative prediction of postoperative recurrence and selection of optimal treatment strategies.

Definitely, this study has some limitations. First, because we could only obtain information about the extent of tissue eosinophilic infiltration from specimens of postoperative patients, all data included only patients who underwent nasal endoscopic surgery, whereas patients with well-controlled CRSwNP without surgical intervention were omitted. Second, although internal validation of the model showed excellent performance, we did not perform prospective external data to verify the generalizability of the model. Finally, because the number of cases of CRSsNP was too sparse, the analysis of its associated subtypes was not included in this study.

In future studies, large, multicenter, and prospective dataset collections are necessary for training and validating AI models. More advanced AI algorithms should also be developed for more efficient and accurate CRS endotype diagnosis, leading the development of a new model of clinical precision diagnosis and treatment of CRS by closely integrating cutting-edge technology AI with the core scientific issues of CRS.

## Figures and Tables

**Table 1:** Performance metrics of single-channel network models versus multichannel network models in validation and test sets.

Dataset	Model	AUC	Accuracy	Sensitivity	Specificity
Training set	Single-channel	0.993	0.993	0.978	0.997
	Multichannel	1.000	1.000	1.000	1.000
Validation set	Single-channel	0.971	0.974	0.954	0.988
	Multichannel	0.980	0.980	0.981	0.980
Test set	Single-channel	0.965	0.967	0.953	0.977
	Multichannel	0.984	0.984	0.981	0.987

**Table 2:** Recognition accuracy of single-channel versus multichannel network models at the image level versus the patient level.

Methods	Model	Accuracy
Image	Single-channel	0.967
	Multichannel	0.984
Patient	Single-channel	0.963
	Multichannel	0.976

## Data Availability

The data that support the findings of this study are available on request from the corresponding authors. The data are not publicly available due to privacy or ethical restrictions.

## References

[1] DeConde A. S., Soler Z. M. (2016). Chronic rhinosinusitis: epidemiology and burden of disease. *American Journal of Rhinology & Allergy*.

[2] Albu S. (2020). Chronic rhinosinusitis—an update on epidemiology, pathogenesis and management. *JCM*.

[3] Shi J. B., Fu Q. L., Zhang H. (2015). Epidemiology of chronic rhinosinusitis: results from a cross-sectional survey in seven Chinese cities. *Allergy*.

[4] Mullol J., Azar A., Buchheit K. M., Hopkins C., Bernstein J. A. (2022). Chronic rhinosinusitis with nasal polyps: quality of life in the biologics era. *The Journal of Allergy and Clinical Immunology: In Practice*.

[5] Fokkens W. J., Lund V. J., Mullol J. (2012). European position paper on rhinosinusitis and nasal polyps 2012. *Rhinology*.

[6] Subspecialty Group of Rhinology, Editorial Board of Chinese Journal of Otorhinolaryngology Head and Neck Surgery and Subspecialty Group of Rhinology, Society of Otorhinolaryngology Head and Neck Surgery, Chinese Medical Association (2019). Chinese guidelines for diagnosis and treatment of chronic rhinosinusitis (2018). *Zhonghua Er Bi Yan Hou Tou Jing Wai Ke Za Zhi*.

[7] Wang E.-T., Zheng Y., Liu P.-F., Guo L.-J. (2014). Eosinophilic chronic rhinosinusitis in East Asians. *World Journal of Clinical Cases*.

[8] Ishitoya J., Sakuma Y., Tsukuda M. (2010). Eosinophilic chronic rhinosinusitis in Japan. *Allergology International*.

[9] Cao P.-P., Li H.-B., Wang B.-F. (2009). Distinct immunopathologic characteristics of various types of chronic rhinosinusitis in adult Chinese. *Journal of Allergy and Clinical Immunology*.

[10] Bachert C., Zhang N., Holtappels G. (2010). Presence of IL-5 protein and IgE antibodies to staphylococcal enterotoxins in nasal polyps is associated with comorbid asthma. *Journal of Allergy and Clinical Immunology*.

[11] Shi L. L., Xiong P., Zhang L. (2013). Features of airway remodeling in different types of C hinese chronic rhinosinusitis are associated with inflammation patterns. *Allergy*.

[12] Wen W., Liu W., Zhang L. (2012). Increased neutrophilia in nasal polyps reduces the response to oral corticosteroid therapy. *Journal of Allergy and Clinical Immunology*.

[13] Payne S. C., Early S. B., Huyett P., Han J. K., Borish L., Steinke J. W. (2011). Evidence for distinct histologic profile of nasal polyps with and without eosinophilia. *The Laryngoscope*.

[14] Cao P.-P., Zhang Y.-N., Liao B. (2014). Increased local IgE production induced by common aeroallergens and phenotypic alteration of mast cells in Chinese eosinophilic, but not non-eosinophilic, chronic rhinosinusitis with nasal polyps. *Clinical & Experimental Allergy*.

[15] Zhu K.-Z., He C., Li Z. (2023). Development and multicenter validation of a novel radiomics-based model for identifying eosinophilic chronic rhinosinusitis with nasal polyps. *Rhinology*.

[16] He S., Chen W., Wang X. (2023). Deep learning radiomics-based preoperative prediction of recurrence in chronic rhinosinusitis. *iScience*.

[17] Georgalas C., Videler W., Freling N., Fokkens W. (2010). Global osteitis scoring scale and chronic rhinosinusitis: a marker of revision surgery. *Clinical Otolaryngology*.

[18] Saito T., Tsuzuki K., Yukitatsu Y., Sakagami M. (2016). Correlation between olfactory acuity and sinonasal radiological findings in adult patients with chronic rhinosinusitis. *Auris Nasus Larynx*.

[19] Bachert C., Marple B., Hosemann W., Cavaliere C., Wen W., Zhang N. (2020). Endotypes of chronic rhinosinusitis with nasal polyps: pathology and possible therapeutic implications. *The Journal of Allergy and Clinical Immunology: In Practice*.

[20] Meng Y., Lou H., Wang C., Luo Z. (2016). Predictive significance of computed tomography in eosinophilic chronic rhinosinusitis with nasal polyps. *In International Forum of Allergy & Rhinology*.

[21] To explore the role of CT scan in the diagnosis of eosinophilic chronic rhinosinusitis. https://www.researchgate.net/publication/325905268_To_explore_the_role_of_CT_scan_in_the_diagnosis_of_eosinophilic_chronic_rhinosinusitis.

[22] Jiang B., Li N., Shi X. (2022). Deep learning reconstruction shows better lung nodule detection for ultra–low-dose chest CT. *Radiology*.

[23] Kim R. Y., Oke J. L., Pickup L. C. (2022). Artificial intelligence tool for assessment of indeterminate pulmonary nodules detected with CT. *Radiology*.

[24] Fokkens W. J., Lund V. J., Hopkins C. (2020). EPOS2020: A Major Step Forward. *Rhinology*.

[25] He K., Zhang X., Ren S., Sun J. (2015). Deep residual learning for image recognition. https://arxiv.org/abs/1512.03385.

[26] Chen X., He K. Exploring simple Siamese representation learning. https://arxiv.org/abs/2011.10566.

[27] Hoffer E., Ailon N. Deep metric learning using Triplet network. https://arxiv.org/abs/1412.6622.

[28] Liu Z., Chen J., Cheng L. (2020). Chinese society of allergy and chinese society of otorhinolaryngology-head and neck surgery guideline for chronic rhinosinusitis. *Allergy, Asthma & Immunology Research*.

[29] Lou H., Wang C., Zhang L. (2019). Endotype-driven precision medicine in chronic rhinosinusitis. *Expert Review of Clinical Immunology*.

[30] Zuo K., Guo J., Chen F. (2014). Clinical characteristics and surrogate markers of eosinophilic chronic rhinosinusitis in southern China. *European Archives of Oto-Rhino-Laryngology*.

